# High-temperature Superconductivity in compressed Solid Silane

**DOI:** 10.1038/srep08845

**Published:** 2015-03-09

**Authors:** Huadi Zhang, Xilian Jin, Yunzhou Lv, Quan Zhuang, Yunxian Liu, Qianqian Lv, Kuo Bao, Da Li, Bingbing Liu, Tian Cui

**Affiliations:** 1 State Key Laboratory of Superhard Materials, College of physics, Jilin University, Changchun, 130012, P. R. China

## Abstract

Crystal structures of silane have been extensively investigated using *ab initio* evolutionary simulation methods at high pressures. Two metallic structures with *P*2_1_/*c* and *C*2/*m* symmetries are found stable above 383 GPa. The superconductivities of metallic phases are fully explored under BCS theory, including the reported *C*2/*c* one. Perturbative linear-response calculations for *C*2/*m* silane at 610 GPa reveal a high superconducting critical temperature that beyond the order of 10^2^ K.

Finding high temperature superconductor is one of the important issues in scientific fields. Since N. W. Ashcroft announced the prediction of the metallic hydrogen^1^, scientists began to explore the hydrogen superconductor. The research about it has never been stopped. Recent theoretical study showed that the *T_c_* of solid metallic hydrogen has achieved 100 K 2. It is still a subject that is full of significant, despite the experimental metallic hydrogen had not found until pressure achieved 300 GPa^3^,^4^.

The hydrogen-rich compounds, especially for the hydrides of group IV, are expected high temperature superconductor for comprehending the superconductivity of metallic hydrogen. This attracted more attention on hydrides of group IV. So far, the theoretical studies of GeH_4_ and SnH_4_ have predicted high superconductivities with maximal *T_c_* reaching 64 K at 220 GPa for GeH_4_^5^ and 62 K at 200 GPa for SnH_4_^6^. In addition, more extensive theoretical and experimental efforts have attempted to reveal the structures and superconductivity of silane. Feng *et al.*^7^ found a *Pman* structure with high superconducting transition temperature *T*_c_ 166 K at 202 GPa. Pickard and Needs^8^ also predicted the structures of SiH_4_ and studied the structural properties, mentioned the possibility of superconductivity in a *C*2/*c* phase. Later Yao *et al*.^9^ showed that the *Pman* structure is in reality not stable by phonon calculations whereas a new *C*2/*c* structure is dynamically stable and the superconductivity is close to 50 K at 125 GPa. Eremets *et al.*^10^ reported that the metallization of silane occurs between 50 and 65 GPa with the *P*6_3_ symmetry and the superconducting transition temperature of 17 K at 96 and 120 GPa by using electrical resistance measurements. Chen *et al.*^11^ demonstrated that the experimental *P*6_3_ metallic structure is dynamically unstable by the phonon calculations, and a new *Cmca* phase was predicted. The *Cmca* silane with a layered network was considered the most likely candidate with the superconducting transition temperature in the range of 20–75 K. Later, Miguel Martinez-Canales *et al.*^12^ found the lower enthalpy structures by using the evolutionary algorithm USPEX than those found in previous studies, and confirmed that SiH_4_ is a low-temperature superconductor with a transition temperature of 17 K. These published theoretical and experimental articles indicate that silane is one of the significant materials for comprehending the superconductivity in metallic hydrogen under high pressures. So far, the high-pressure structures of silane are still in discussion, and the expected high-temperature superconductivity similar with metallic hydrogen has not been reported. In this paper, the high-pressure crystal structures and potential superconducting property of silane have been extensively explored. Two metallic structures with space groups *P*2_1_*/c* and *C*2*/m* have been found stable from 383 to 606 GPa and above 606 GPa respectively. Further calculations discover that the superconducting transition temperature has more than 100 K for the *C*2/*m* phase. In addition, the superconductivity of *C*2/*c*^8^ has been deeply explored in our work.

## Results and Discussion

**Figure 1:**
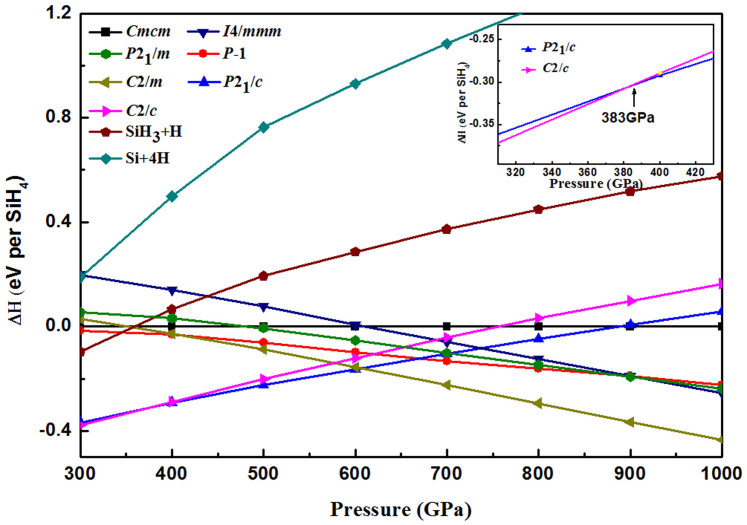
Enthalpy difference curves of Silane. Calculated enthalpies per SiH_4_ unit of various structures relative to our predicted *Cmcm* structure as a function of pressure range from 300–1000 GPa. Inset: Enthalpies in the pressure range from 310 GPa to 430 GPa.

**Figure 2:**
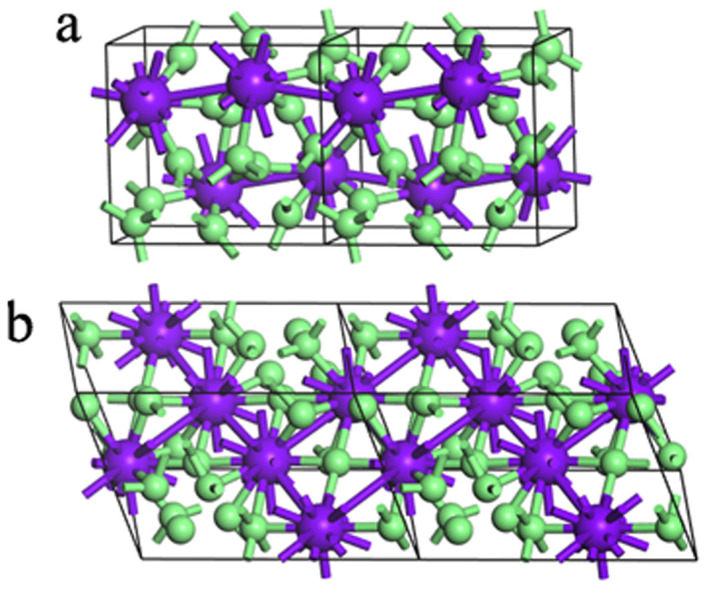
Structures of silane. Big atoms depict Si, while small atoms represent H. (a) Conventional cell of *P*2_1_/*c* silane, stable between 383 and 606 GPa. (b) Primitive cell of *C*2/*m* silane, favored above 606 GPa.

The crystal structures of silane were predicted with one to six SiH_4_ formula units per cell. Several competitive candidates for energy with the space groups *C*2/*c* (2 molecules/cell), *Cmcm* (2 molecules/cell), *I*4/*mmm* (3 molecules/cell), *P*2_1_/*c* (4 molecules/cell), *P*2_1_/*m* (4 molecules/cell), *P*-1 (4 molecules/cell) and *C*2/*m* (6 molecules/cell) were obtained. The enthalpies of candidates are plotted as a function of pressures in Figure 1. Above the 300 GPa, there are two competitive enthalpy structures with the *C*2/*c* and *P*2_1_/*c* symmetry. The maximum enthalpy difference between them is only 0.01 (eV/unit cell) over the pressure range from 300 GPa to 383 GPa, see Figure 1 inset. The *C*2/*c* phase predicted in this work is identical with the one proposed by C. J. Pickard and R. J. Needs^8^, which forms three-dimensional networks. Above 383 GPa, *P*2_1_/*c* phase take over *C*2/*c* phase and becomes most competitive on enthalpy. As shown in Figure 2(a), Si atoms show a fold layered arrangement with H atoms site around them. There are four SiH_4_ units with five unequivalent atoms in the conventional cell. One Si atom and four H atoms occupy the crystallographic 4*e* position with 1 symmetry in this monoclinic crystal. The shortest distance between H atoms is 1.045 Å at 400 GPa, which is longer than the 0.762 Å of the H-H bond length in “H_2_” unit in the *Cmca-12* structure^13^ of solid hydrogen at the same pressure. *P*2_1_/*c* phase keep stable on enthalpy at least to 606 GPa until another competitive phase with *C*2/*m* symmetry appears, as shown in Figure 1. *C*2/*m* phase obtains monoclinic base-centered lattice, and contains six formula units in the conventional cell. There are three unequivalent Si atoms occupy the 4*i* position with m symmetry, whereas nine H atoms sit on the 8*j* (site symmetry is 1), 4*i* (site symmetry is m) and 4*g* (site symmetry is 2) positions respectively. The shortest distance between two H atoms is 1.009 Å, and slightly shorter than 1.045 Å in the *P*2_1_/*c* phase. The coordination of Si atoms in *C*2/*c*, *P*2_1_/c, and C2/m are all eleven, *i.e.* each Si atom bond with eleven H atoms, which is quite different with the molecule crystal under low pressure^14^ and implies some different physical characters. Detail parameters of the structures are listed in Table 1. The decomposition enthalpies reference to *Cmca*-12^13^ structure of H_2_ (below 500 GPa), *I*4_1_/*amd*^13^,^15^ structure of H_2_ (500–1000 GPa), *Fm*-3*m*^16^ structure of Si and *C*2/*c*^17^ structure of SiH_3_ can also be seen in Figure 1 (The comparison with SiH_4_(H_2_)_2_ are shown in supplementary information). It is noteworthy that the enthalpies of the three structures are lower. Moreover, *P*2_1_/*c* and *C*2/*m* structures are found to be energetically much superior to previous structures^7^,^8^,^9^,^11^,^12^. Therefore, three monoclinic (*C*2/*c*, *P*2_1_/c, and *C*2/*m*) phases can be taken as energetically stable structures of silane under high pressure range, see Figure 1.

**Figure 3:**
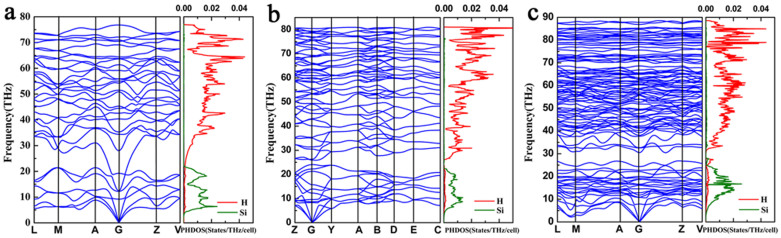
The phonon band structure and projected phonon DOS charts. (a) *C*2/*c* phase at 300 GPa. (b) *P*2_1_/*c* phase at 400 GPa. (c) *C*2/*m* phase at 610 GPa.

The mechanical stability of structure can provide insight into the stability of materials. To evaluate the mechanical stability of the *C*2/*c*, *P*2_1_/*c* and *C*2/*m* phases, elastic constants have been calculated and listed in Table 2. According to the mechanical stability criteria, the crystal deformation energy is positive, this means the determinants of elastic constants matrix *C_ij_* should be positive^18^. Considering the crystal symmetry, the mechanical stability of expression will be further simplified^19^. It can be found that the elastic constants of these three structures satisfy the mechanical stability criteria, indicating that these three structures are mechanically stable. The phonon band structure and projected phonon density of states (PHDOS) of the three phases at selected pressures are presented in Figure 3. Absence of any imaginary frequency in the Brillouin zone establishes the dynamical stability. The PHDOS of these three structures shows that the heavier Si atoms dominate the low-frequency vibrations, and the lighter H atoms contribute significantly to the high-frequency modes.

**Figure 4:**
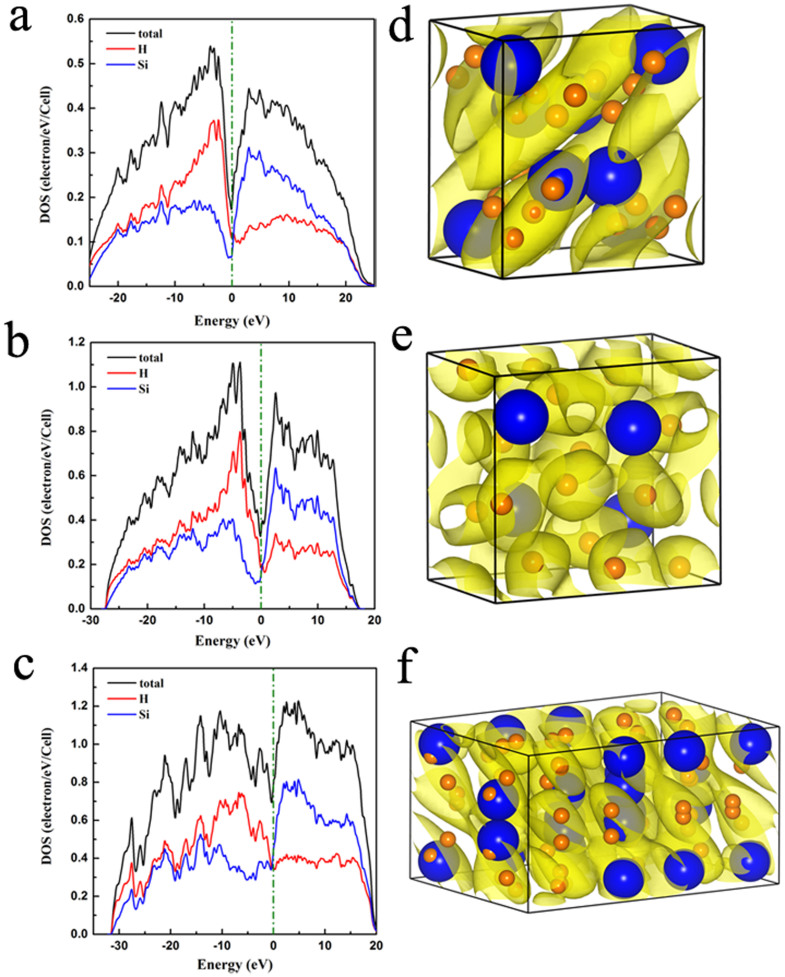
The electronic projected DOS and electron localization function isosurface maps. Electronic projected DOS of (a) *C*2/*c* phase at 300 GPa, (b) *P*2_1_/*c* phase at 400 GPa, and (c) *C*2/*m* phase at 610 GPa, respectively. Electron localization function isosurface maps for (d) *C*2/*c* structure at 300 GPa, (e) *P*2_1_/*c* structure at 400 GPa, (f) *C*2/*m* structure at 610 GPa. The yellow isosurface represents an ELF value of 0.5. Blue and orange spheres represent Si and H, respectively.

To analysis the electronic properties of *C*2/*c*, *P*2_1_/*c* and *C*2/*m* phases, we first calculated the electronic density of states (DOS). As shown in Figure 4, they are all metals with large total DOS at Fermi level. Specially, for *C*2/*m* structure, the total DOS of Fermi level is significantly higher than the other structures. These high DOS values might favor the superconducting behavior. From the Figure 4(a–c), H atoms contribute more to DOS than the Si atoms below Fermi level, and contribute less to DOS than the Si atoms above Fermi level. With increasing pressure, the contributions from atoms H and Si do not significantly change. The electron localization functions (ELF)^20^ of the *C*2/*c*, *P*2_1_/*c*, and *C*2/*m* phases are calculated at 300 GPa, 400 GPa, and 610 GPa, respectively. The isosurface plots at ELF = 0.5 are shown in Figure 4(d–f). The electron-gas-like distribution in this space are connected, which conforms the three structures are metallic.

**Figure 5:**
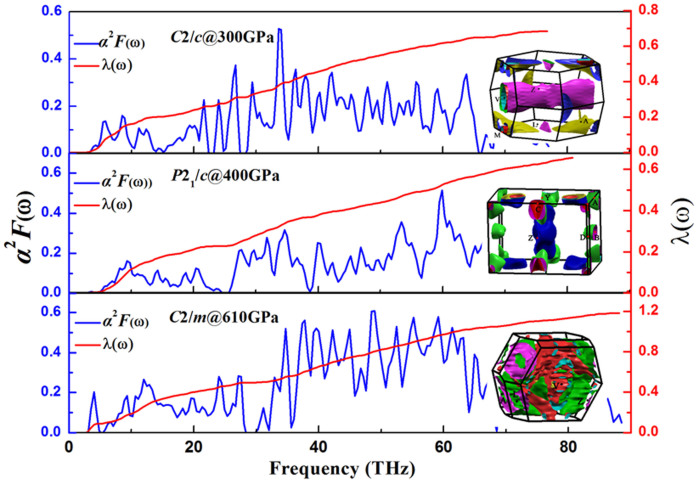
The Eliashberg phonon spectral function *α^2^F(ω)*, the electron-phonon integral *λ* and the three-dimensional Fermi surface of *C*2/*c*, *P*2_1_/*c* and *C*2/*m* at 300, 400, and 610 GPa, respectively.

The electron-phonon coupling strength (EPC) *λ* and the logarithmic average phonon frequency *ω*_log_ of the three structures were calculated to explore the possible superconductivity of SiH_4_. The Eliashberg phonon spectral function *α^2^F*(ω) and the *λ* as a function of frequency are shown in Figure 5. The *λ* of *C*2/*c* structure at 300 GPa, *P*2_1_/*c* structure at 400 GPa and *C*2/*m* structure at 610 GPa are 0.69, 0.66 and 1.18, respectively. The superconducting critical temperature can be estimated from the Allen–Dynes modified McMillan equation^21^
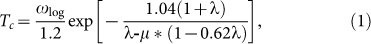
which has been found to be highly accurate for many materials with *λ* < 1.5. The Coulomb pseudopotential μ* is taken as 0.13 for hydrogen dominant metallic alloys by Ashcroft^22^, resulting that the estimated *T*_c_ of *C*2/*c*, *P*2_1_/*c* and *C*2/*m* at 300 GPa, 400 GPa, and 610 GPa are 29.65 K, 31.57 K and 106.31 K, respectively. Subsequently, the contributions to EPC *λ* of each atom are analyzed. As for *C*2/*c*, *P*2_1_/*c* and *C*2/*m*, Si vibrations provide a contribution of 36%, 34%, and 37% respectively, while the H translational vibrations contribute for nearly 64%, 66% and 63% respectively. The result shows that the element H plays a significant role in the EPC *λ*.

**Figure 6:**
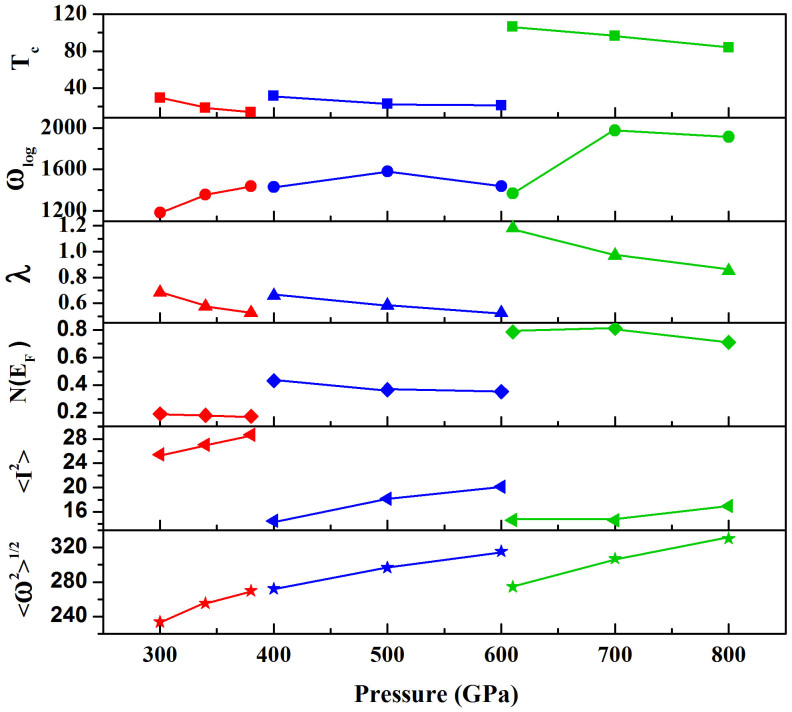
Calculated *Tc* (K), the logarithmic average phonon frequency *ω*_log_ (K), electron-phonon coupling strength *λ,* the DOS at the Fermi level N(ϵ_F_) (state/eV/Cell), the square of the electron-ion matrix element 〈*I*^2^〉 (eV/Å)^2^ and the average phonon frequency 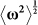 (THz) of the three phases as a function of pressure. The red line respect *C*2/*c* phase at 300–380 GPa. The blue line respect *P*2_1_/*c* phase at 400–600 GPa. The green line respect *C*2/*m* phase at 610–800 GPa.

The trend of *T_c_* with pressures for these three structures was explored for further investigation. *T_c_*, *ω*_log_ and *λ* along with the changes of pressures are displayed in Figure 6. With increasing pressure, the decrease of *λ* plays an important role to the downward trend of *T*_c_. Similar phenomena can be observed in other hydrogen-rich materials^10^,^23^,^24^,^25^. We then use the rigid-muffin-tin (RMT) theory of Gaspari and Gyorffy^26^ for further analysis this variation. MaMillan's strong coupling theory^22^,^27^ defines an electron-phonon coupling constant by
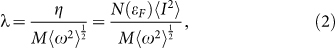
where *M* is the atomic mass. The DOS at the Fermi level *N*(*ϵ_F_*), the square of the electron-ion matrix element 〈*I*^2^〉 and the average phonon frequency 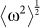 of the three phases at several pressures are shown in Figure 6. From the Figure, 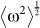 increases generally with pressure, meanwhile *N*(*ϵ_F_*) decrease (the values between *C*2/*m* at 610 GPa and 700 GPa are closely identical). The tendency of the two parameters made *λ* become lower and lower, which lead to the decrease of *T*_c_ with increasing pressure.

We also realized that *T*_c_ magnitude of *C*2/*m* is beyond 10^2^ K which is higher than other two phases that promoted us to explore the underlying superconducting mechanism. From Figure 6, the trend of *λ* along with pressures is consistent with *T*_c_. So, *λ* played an important role in the superconducting critical temperature. The calculated EPC *λ* of *C*2/*m* phase at 610 GPa is 1.18, which is much higher than the values of 0.69 and 0.66 of two phases. The bigger EPC *λ* can directly contribute to higher *T*_c_. Furthermore, formula (2) defined by MaMillan's strong coupling theory can be used to analyze the mechanisms. Comparing the contribution to *λ* in the phase of *C*2/*m* and *C*2/*c*, Figure 6 reveals that *N*(*ϵ_F_*) of *C*2/*m* contributes about four times than the one of *C*2/*c*, meanwhile the two factors of 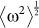 and 〈*I*^2^〉 together contribute about half of *C*2/*c*. Although there are two disadvantages of parameters in *C*2/*m* according formula (2), the higher *N*(*ϵ_F_*) sustains the higher *λ* as a whole. As for *C*2/*m* and *P*2_1_/c, there are no obvious differences on the values of 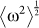 and 〈*I*^2^〉, and *λ* can be dominated by *N*(*ϵ_F_*) as well. As shown in Figure 6, *N*(*ϵ_F_*) in *C*2/*m* is much higher than the ones in other two structures. Based on an overall analysis of contributions from parameters in two formulas including the contribution from *ω*_log_, *N*(*ϵ_F_*) play an important role to the much higher *T_c_* than other phases.

It is consistent with the calculated three-dimensional Fermi surface which is shown in Figure 5. There are four electronic bands across the Fermi surface for all the three phases, and identified by different colors. As shown in Figure 5, the *C*2/*m* phase is filling with much more Fermi surfaces in the brillouin zone than the two other phases, which is corresponding with the high level of *N*(*ϵ_F_*). Therefore, the Fermi surface also support the conclusion that the high values of *N*(*ϵ_F_*) is significant to sustain high *T_c_*.

## Conclusion

In summary, we have extensively investigated crystal structures and superconductivity of silane. The *P*2_1_/*c*, and *C*2/*m* structures are found which are thermodynamically, mechanically, and dynamically stable. The superconducting critical temperature *T*_c_ of the *C*2/*c* phase at 300 GPa and *P*2_1_/*c* at 400 GPa are 29.65 K and 31.57 K. The superconductivity of the *C*2/*m* phase with a transition temperature 106.31 K is found to be mainly attributed to the strong electron-phonon coupling due to the high electronic density of states at the Fermi level.

## Methods

The most stable structures of silane under high pressures were performed using evolutionary algorithm, as implemented in the USPEX code^28^. This approach has been successfully used in the study of many materials at high pressures^29^,^30^. The structural relaxations have been performed within the framework of the generalized gradient approximation (GGA)^31^ with the Perdew–Burke–Ernzerhof parameterization for the exchange-correlation functional to DFT by using the projector augmented-wave(PAW) method^32^, as implemented in *ab initio* VASP code^33^. The structures were relaxed at a high cutoff energy of 1000 eV, and a Brillouin zone sampling grid of spacing 2π × 0.03 Å^−1^. The lattice dynamics and electron-phonon coupling have been computed with QUANTUM-ESPRESSO^34^. We used Vanderbilt-type ultrasoft pseudopotentials^35^ with a cutoff energy of 40 Ry. Phonon frequencies were calculated based on the density functional linear-response method^36^. A Monkhorst-Pack(MP)^37^ Brillouin zone sampling grid of spacing 2π × 0.025 Å^−1^ with Gaussian smearing of 0.02 Ry were used for the phonon calculations, at 4 × 4 × 4, 5 × 4 × 3 and 3 × 3 × 2 *q*-point mesh for *C*2/*c*, *P*2_1_/*c* and *C*2/*m* for the electron-phonon interaction matrix element, respectively. All the convergences of the plane-wave basis set and MP sampling are carefully examined by employing higher kinetic energy cutoffs and denser grids sets. The tests of cutoff energy and the validity of potential functions are shown in supplementary information.

## Author Contributions

T.C. initiated the project. H.Z. performed the first principle calculations and prepared all figures. H.Z., X.J. and T.C. analyzed the data and wrote the manuscript text. Y.Z.L., Q.Z., Y.X.L., Q.L., K.B., D.L. and B.L. reviewed the manuscript.

## Supplementary Material

Supplementary Information Supplementary information

## Figures and Tables

**Table 1:** Structural parameters for the favored structures suggested in this work

Space group # atoms # Pressure	Lattice parameters (Å, °)	Atomic coordinates (fractional)				Site
*P*2_1_/c	a = 2.7260	H1	0.33186	0.54676	0.91785	4e
400 GPa	b = 3.8882	H2	0.54601	0.83973	0.08510	4e
	c = 4.2525	H3	0.65917	0.37847	0.73239	4e
	β = 88.2231	H4	0.17665	0.91106	0.08146	4e
		Si1	0.86906	0.70548	0.84406	4e
*C*2*/m*	a = 6.7297	H1	0.52517	0.30530	0.76854	8j
610 GPa	b = 3.0948	H2	0.22275	0.19521	0.65601	8j
	c = 6.0792	H3	0.22252	0.80696	0.85411	8j
	β = 63.4329	H4	0.87273	0.00000	0.94004	4i
		H5	0.37413	0.00000	0.68966	4i
		H6	0.62206	0.00000	0.81435	4i
		H7	0.11526	0.00000	0.82576	4i
		H8	0.12208	0.00000	0.55910	4i
		H9	0.50000	0.31851	0.00000	4g
		Si1	0.87715	0.00000	0.67939	4i
		Si2	0.61367	0.00000	0.56500	4i
		Si3	0.37196	0.00000	0.94522	4i

**Table 2:** Elastic constants *C_ij_* (GPa) of *C*2/c, *P*2_1_/c, and *C*2*/m* crystals calculated at 300, 400, and 610 GPa, respectively

	*C*_11_	*C*_22_	*C*_33_	*C*_44_	*C*_55_	*C*_66_	
	*C*_13_	*C*_15_	*C*_23_	*C*_25_	*C*_35_	*C*_46_	*C*_12_
*C*2/*c* (300 GPa)	1285.47	1117.27	1407.65	408.27	535.20	242.69	948.40
	578.56	118.06	796.25	64.00	−146.20	114.85	
*P*2_1_/*c* (400 GPa)	1590.95	1627.44	1816.35	396.65	387.36	224.65	1072.97
	945.00	34.90	760.58	−29.57	4.02	−72.45	
*C*2/*m* (610 GPa)	2102.20	2227.44	2234.93	392.92	483.57	422.65	1385.08
	1553.58	−93.95	1256.96	−50.93	97.95	−2.67	
