# Supplementary material for: High-temperature Superconductivity in compressed Solid Silane
Source: Sci Rep. 2015 Mar 9;5:8845. doi: 10.1038/srep08845 (PMC4352889; doi:10.1038/srep08845)
Supplement: Supplementary Information [file srep08845-s1.pdf]

Supplementary information

High-temperature Superconductivity in compressed Solid Silane

Huadi Zhang, Xilian Jin, Yunzhou Lv, Quan Zhuang, Yunxian Liu, Qianqian Lv, Kuo Bao, Da Li, Bingbing Liu, Tian Cui*

State Key Laboratory of Superhard Materials, College of physics, Jilin University, Changchun, 130012, P. R. China

1. Convergence test

Convergence test is very significant before calculating the properties. The lattice dynamics and electron-phonon coupling have been computed by QUANTUM-ESPRESSO with cutoff energy of 40 Ry in our paper. Base on the testing results, we find 40 Ry is enough to achieve the convergence precision, and is sufficient for accurate calculations of properties. Take for example of *C*2/*c* phase, the calculated energies per atom as a function of different cutoff energies are shown in Figure S1. From the figure, the difference energy per atom between 40 Ry and 100 Ry is only 0.00045 eV/atom. So, we think the total energy has been achieved convergence with 40 Ry. Moreover, the other two structures also have been tested, and show the same result with the one of *C*2/*c*.

Furthermore, we use the cutoff energy 80 Ry instead of 40 Ry to relax the structure and calculate the superconducting translation temperature. Take for example of *C*2/*c* phase, the T_c at 300 GPa is 29.68 K, which is very close to 29.65 K

with cutoff energy 40 Ry. So, we think that 40 Ry is well enough for calculating the properties.

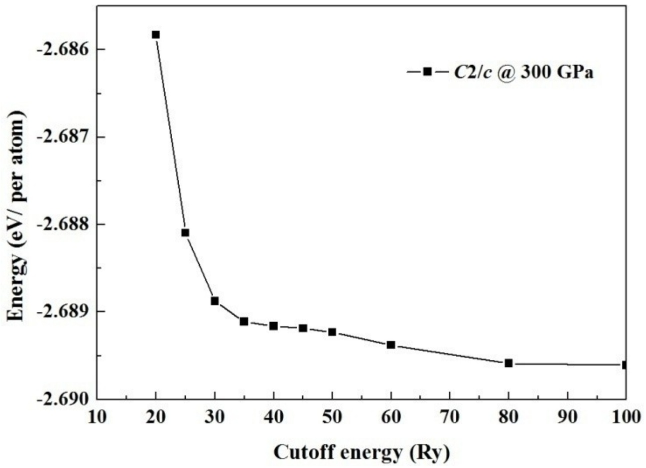

Figure S1 Different energy as a function of cutoff energy for *C*2/*c* phase at 300 GPa.

2. Potential functions test

The potential functions are usually important and have a distinct impact on the result. In this paper, PAW_PBE potential which are named H_h, Si_h in the VASP library were selected. H_h and Si_h are more hard potentials in which the cutoff radius equals 0.42 Å and 0.79 Å. To test the H potential function's validity, H_AE named in the VASP library was used. H_AE is a special Coulomb potential with 1/r form, which can construct accurate results up to TPa. The equations of state (EOS) of high pressure phase (>500 GPa) $I4_1/amd$ in H2 using H_h and H_AE are shown in Figure S2 (a). Two curves are almost indistinguishable in pressure range 500 – 1000 GPa, proving H_h is well used in our paper. The high-pressure phase of Si is *Fm*-3*m*.

To test the Si potential function's validity, the EOS of using Si_h potential function and all-electron full-potential function were calculated. The EOS of all-electron full-potential function was calculated by an all-electron full-potential linearised augmented plane-wave (FP-LAPW) code ELK[1]. The result is shown in Figure S2 (b). Compared with all-electron full-potential function, Si_h agrees well. The max difference per atom is only 3.9% in the pressure range 500 – 1000 GPa that testified Si_h is suitable used above 500 GPa in our work. Furthermore, the shortest H-H bond length, Si-H bond length and Si-Si bond length of the stable structures in our work is always greater than the corresponding cutoff radius in potential functions'. Therefore, the PAW potentials in our paper are suitable up to 1000 GPa.

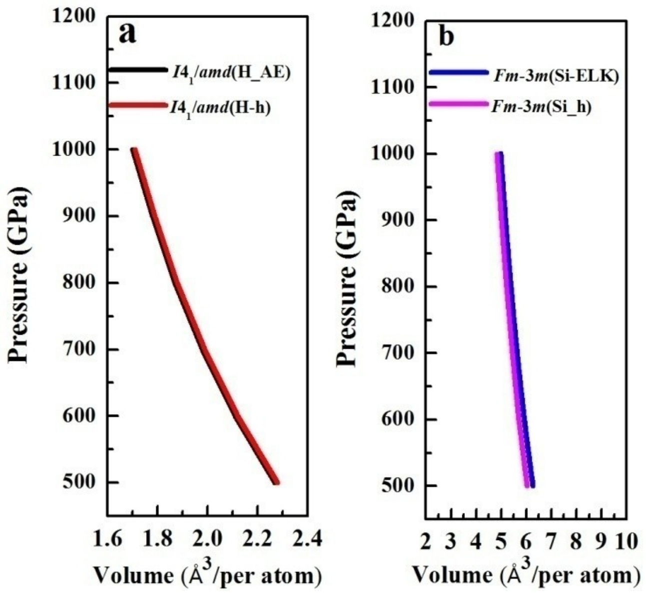

Figure S2 The calculated EOSs. (a) The EOSs of the high-pressure phase $I4_1/amd$ in solid hydrogen with different PAW potentials from 500 to 1000 GPa. (b) The EOSs of the high-pressure phase *Fm*-3*m* in Si from 500 to 1000 GPa.

3. Thermodynamic stability

It is important to know the compositional stability. We have considered all possible compounds that have been predicted by theory or have been confirmed by experiment in Si-H system. For $SiH_4(H_2)_2$[2], the enthalpy differences between $SiH_4(H_2)_2$ and our structures are shown in the Figure S3. The structure of $SiH_4(H_2)_2$ is *Ccca* in pressure above 248 GPa by Li *et al.* H_2 is reference to *Cmca*-12 structure (below 500 GPa) and $I4_1/amd$ (500-1000 GPa). From the figure, the enthalpy of $SiH_4(H_2)_2$ is higher than the ones of our structures through the pressure range from 300 - 1000 GPa. What's more, the *Ccca* phase of $SiH_4(H_2)_2$ has been distorted around 800 GPa. The symmetry has been changed, leading to the *Ccca* phase no longer exist in our higher pressure range.

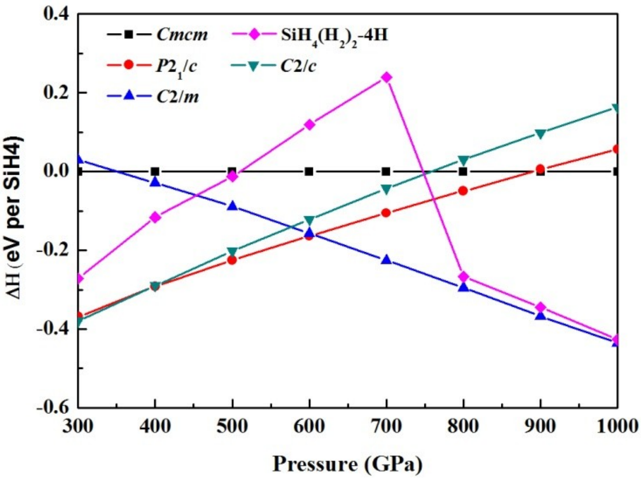

Figure S3 Enthalpy differences between $SiH_4(H_2)_2$ and our structures.

Reference

1. The ELK FP-LAPW Code. http://elk.sourceforge.net.
2. Li, Y. *et al*. Superconductivity at ~100K in dense $SiH_4(H_2)_2$ predicted by first principles. *Proc. Natl. Acad. Sci. U.S.A.* **107**, 15708-15711 (2010).
